# Recent Advances in Metabolic Engineering, Protein Engineering, and Transcriptome-Guided Insights Toward Synthetic Production of Taxol

**DOI:** 10.3389/fbioe.2021.632269

**Published:** 2021-02-05

**Authors:** Ishmael Mutanda, Jianhua Li, Fanglin Xu, Yong Wang

**Affiliations:** ^1^Key Laboratory of Synthetic Biology, CAS Center for Excellence in Molecular Plant Sciences, Institute of Plant Physiology and Ecology, Chinese Academy of Sciences, Shanghai, China; ^2^University of Chinese Academy of Sciences, Beijing, China; ^3^Key Laboratory of Plant Stress Biology, State Key Laboratory of Cotton Biology, School of Life Sciences, He’nan University, Kaifeng, China

**Keywords:** Taxol, protein engineering, transcriptome, paclitaxel, taxadien-5α-ol, taxane-5α-hydroxylase, metabolic engineering

## Abstract

The diterpenoid paclitaxel (Taxol^®^) is a blockbuster anticancer agent that was originally isolated from the Pacific yew (*Taxus brevifolia*) five decades ago. Despite the wealth of information gained over the years on Taxol research, there still remains supply issues to meet increasing clinical demand. Although alternative Taxol production methods have been developed, they still face several drawbacks that cause supply shortages and high production costs. It is highly desired to develop biotechnological production platforms for Taxol, however, there are still gaps in our understanding of the biosynthetic pathway, catalytic enzymes, regulatory and control mechanisms that hamper production of this critical drug by synthetic biology approaches. Over the past 5 years, significant advances were made in metabolic engineering and optimization of the Taxol pathway in different hosts, leading to accumulation of taxane intermediates. Computational and experimental approaches were leveraged to gain mechanistic insights into the catalytic cycle of pathway enzymes and guide rational protein engineering efforts to improve catalytic fitness and substrate/product specificity, especially of the cytochrome P450s (CYP450s). Notable breakthroughs were also realized in engineering the pathway in plant hosts that are more promising in addressing the challenging CYP450 chemistry. Here, we review these recent advances and in addition, we summarize recent transcriptomic data sets of *Taxus* species and elicited culture cells, and give a bird’s-eye view of the information that can be gleaned from these publicly available resources. Recent mining of transcriptome data sets led to discovery of two putative pathway enzymes, provided many lead candidates for the missing steps and provided new insights on the regulatory mechanisms governing Taxol biosynthesis. All these inferences are relevant to future biotechnological production of Taxol.

## Introduction

The blockbuster antitumor drug paclitaxel (Taxol^®^) is a highly functionalized plant diterpenoid discovered in the late 1960s in pacific yew (*Taxus brevifolia*) plants. Though it went through a long developmental phase, it later became the most successful plant natural product in use as an effective chemotherapy drug since its initial Food and Drug Administration (FDA) approval in 1992 (Wani et al., 1971; Wani and Horwitz, 2014). Taxol was initially approved for treatment of refractory ovarian cancer and breast carcinomas, but over the years it has found expanded clinical uses in treatment of non-small cell lung carcinoma, Kaposi’s sarcoma and cancers of the lung, breast, bladder, prostate, esophagus and pancreas when used alone or in combination therapies with other antineoplastic agents.

Original production process involved direct isolation from the bark of yew plants, but the process is very destructive and inefficient, requiring three trees (∼12 kg bark material) to produce 1 g pure Taxol or ca. 3,000 yew trees to produce only 1 kg of the drug (Malik et al., 2011; McElroy and Jennewein, 2017). To add to this, Taxol is produced as a complex mixture with hundreds of other taxanes in *Taxus* plant tissue, making the purification of this highly hydrophobic compound very laborious and environmentally damaging due to use of organic solvents (Wani and Horwitz, 2014). Total chemical synthesis routes to Taxol were developed (Holton et al., 1994; Nicolaou et al., 1994), but are not commercially viable owing to the many steps required and cost considerations. Inspired by the success of Taxol, and the need to address supply and ecological challenges, alternative production platforms have been developed: (i) extraction of 10-deacetylbaccatin III (10-DAB) or baccatin III (BIII) from renewable twigs of *Taxus* species, that can be semi-synthetically modified to Taxol, and (ii) plant cell fermentation (PCF) involving use of *Taxus* cell suspension cultures (Fett-Netto et al., 1992; Mountford, 2010; Malik et al., 2011).

Despite several optimization efforts to improve these methods, they still suffer from several drawbacks that limit their capacity and also drive the price of Taxol high; (i) production from twigs is still dependent on yew trees, thus is susceptible to weather and environmental factors, (ii) long maturity time of the trees and (iii) extraction process still involves use of organic solvents. The PCF route presents obvious advantages over direct extraction from field-grown *Taxus* twigs, but it suffers from its own shortcomings; (i) instability of cell lines in the long fermentation periods (ii) use of endogenous biosynthetic pathways, thus no genetic engineering targets to improve flux toward Taxol and (iii) poor Taxol yields in the fermenters, even with elicitors (Choi et al., 2000; Ketchum and Croteau, 2006).

**FIGURE 1:**
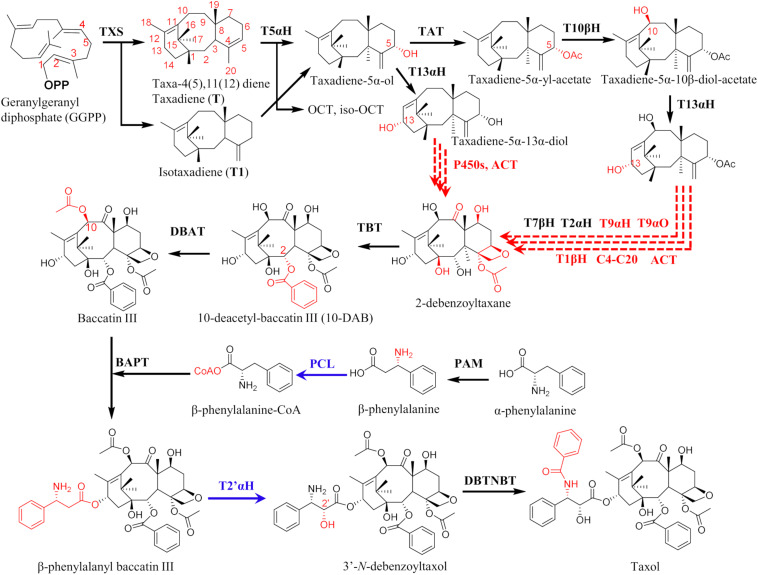
Taxol biosynthetic pathway from geranylgeranyl diphosphate, showing carbon positions. Enzymes in red are not yet characterized and steps in red dotted arrows are not yet fully elucidated. Blue arrows—genes encoding putative PCL and T2′αH were recently isolated, though not yet applied in metabolic engineering designs. Enzyme abbreviations: TXS, taxadiene synthase; T5αH, taxane-5α-hydroxylase; TAT, taxane-5α-ol-O-acetyltransferase; T10βH, taxane-10β-hydroxylase; T13αH, taxane-13α-hydroxylase; T1βH, taxane 1β-hydroxylase; T9αH, taxane 9α-hydroxylase; T9αO, taxane 9α-dioxygenase; T2αH, taxane 2α-hydroxylase; T7βH, taxane 7β-hydroxylase; C4-C20, C4-C20 epoxidase; TBT, taxane-2α-O-benzoyl transferase; DBAT, 10-deacetylbaccatin III-10-O-acetyltransferase; PAM, phenylalanine aminomutase; PCL, phenylalanine-CoA ligase; BAPT, C-13 phenylpropanoyl-CoA transferase; T2′αH, taxane 2′α-hydroxylase; DBTNBT, Debenzoyl taxol *N*-benzoyl transferase. Products of T5aH—OCT, 5(12)-oxa-3 (11)-cyclotaxane and iso-OCT, 5(13)-oxa-3(11)-cyclotaxane, ACT, acyl-CoA transferase.

For these reasons, it is highly desirable to establish biotechnological production systems for Taxol production that will address most of these issues. Production in more amenable, fast-growing heterologous hosts offers more advantages in terms of control and manipulation of metabolic flux by improving enzyme expression, pathway regulation, availability of cofactors and engineering competing pathways. However, there are challenges that prevent development of desired sustainable Taxol biotechnological platforms: the biosynthetic pathway is long and complicated, involving 19 expected steps from geranylgeranyl diphosphate (GGPP) the universal precursor of diterpenoids (Figure 1) and there are still many gaps in our understanding of Taxol biosynthesis and its regulatory mechanisms. Several cytochrome P450 (CYP450) hydroxylases and an epoxidase remain missing (Figure 1). Even for the steps with known enzymes, expression of functional pathway enzymes in heterologous hosts, especially the CYP450s is proving to be non-trivial.

In this review, we discuss various strategies conducted in the last 5 years to develop heterologous systems for Taxol biosynthesis. Building from the breakthrough strategy 10 years ago (Ajikumar et al., 2010), recent advances in synthetic biology have been applied in several studies to optimize pathway expression, develop enzyme screening platforms and engineer pathway enzymes to get variants with improved catalytic performance and higher specificity. We also highlight the use of computational tools that were leveraged in gaining insights in the catalytic mechanisms of several enzymes and lastly, we summarize recent Taxol-related transcriptomes and how this publicly available resource was recently used in inferring regulatory mechanisms, mining the missing genes and providing many lead candidates for missing steps. In the coming years, we expect this knowledge to be pivotal in development of biotechnological systems for production of this critical drug.

## Challenges With Metabolic Engineering of Taxol Pathway

Metabolic engineering of the Taxol pathway in heterologous hosts is highly desirable as it will establish a versatile, direct route to the critical anticancer drug. Biosynthetic production systems are expected to improve supply of Taxol, as well as lower the production costs through improved efficiency and use of inexpensive sugars as starting material in microbial factories. Another potential avenue under exploration is production in cheaper, high biomass and fast-growing plants like tobacco that can be engineered for high-level production of non-native metabolites using carbon resources from photosynthesis. Before realizing this dream, there are several challenges with Taxol biosynthesis that should be addressed.

### The Missing Pathway Enzymes

Taxol is derived from the C_5_ terpenoid universal precursors, dimethylallyl diphosphate (DMAPP) and isopentenyl pyrophosphate (IPP) through geranylgeranyl diphosphate (GGDP), the C_20_ universal precursor of all diterpenoids. The structure of the Taxol molecule is complex and likewise, the biosynthetic pathway is equally complex; five decades after its discovery, the pathway is not yet fully understood, with several steps still undefined, and several enzymes still missing. These gaps in our understanding of the biosynthetic pathway deprive us of the critical framework to manipulate Taxol biosynthesis and develop heterologous production systems. The pathway is hypothesized to involve 19 steps from GGPP (Figure 1), involving terpene cyclization, 9 cytochrome P450-catalyzed hydroxylations, 3 acylations, acetylations, oxetane ring formation, benzoylations, and phenylisoserine side chain attachment. Exceptional work in the last 25 years has contributed genes and enzymes of up to 14 steps; from taxadiene synthase (TXS) that catalyzes the first and committed step to cyclize GGPPS to taxadiene (Hezari et al., 1995; Wildung and Croteau, 1996), five CYP450s [taxane 5α-hydroxylase (T5αH), taxane 2α-hydroxylase (T2αH), taxane 7β-hydroxylase (T7βH), taxane 10β-hydroxylase (T10βH) and taxane 13α-hydroxylase (T13αH)] (Hefner et al., 1996; Jennewein et al., 2001; Kaspera and Croteau, 2006; Schoendorf et al., 2001; Walker et al., 2000; Walker and Croteau, 2001), five acyl CoA transferases (ACTs) [taxane-5α-ol-O-acetyltransferase (TAT), taxane-2α-O-benzoyl transferase (TBT), 10-deacetylbaccatin III-10-O-acetyltransferase (DBAT), C-13 phenylpropanoyl-CoA transferase (BAPT) and Debenzoyl taxol *N*-benzoyl transferase (DBTNBT)] and phenylalanine aminomutase (PAM).

A putative ACT that activates β-phenylalanine to β-phenylalanine-CoA was isolated from *T. baccata* cell cultures (Ramírez-Estrada et al., 2016), and was identified as β-phenylalanine-CoA ligase (PCL) after functional characterization. However, a recently isolated homolog of this PCL displaying 99% sequence identity showed no detectable activity against both β-phenylalanine and 3-phenylisoserine *in vitro* (Srividya et al., 2020), thus, the suitability of this clone for heterologous expression in other hosts might remain an open question. Other still missing enzymes are the CYP450s taxane 1β-hydroxylase (T1βH), taxane 9α-hydroxylase (T9αH), taxane 9α-oxidase (T9αO), and C4,5 epoxidase (Figure 1). Taxane 2′α-hydroxylase (T2′αH) was recently isolated from mining *T. baccata* transcriptome (Sanchez-Muñoz et al., 2020), and the details are reviewed in the section on transcriptomes below. The missing enzymes, together with other challenges outlined below need to be resolved before successful production of Taxol by synthetic biology systems. Other enzymes have been identified that channel flux toward off-pathway intermediates that do not lead to Taxol, such as the taxane 14β-hydroxylase (T14βH), the recently discovered ACTs that attach different groups to 3′-*N*-debenzoyltaxol (Srividya et al., 2020) and many more that are expected considering the chemical diversity of taxanes in plant tissues.

### Poor Expression of Functional Enzymes and Challenges in CYP450 Chemistry

Oxygenation chemistry of Taxol presents a formidable barrier to both pathway elucidation and metabolic engineering efforts, starting with the first hydroxylation of the C5 position of taxadiene by T5αH (Hefner et al., 1996; Jennewein et al., 2004). All *Taxus* CYP450s identified and functionally characterized for activity in Taxol biosynthesis are from the CYP725A subfamily, including T5αH (CYP725A4). T5αH accepts both taxa-4(5),11(12) diene (taxadiene, **T**) and its close alternative isomer taxa-4(20),11(12) diene (isotaxadiene, **T1**), that are both products of TXS to form taxadiene-5α-ol (T-5α-ol) (Figure 1). This CYP450 presents several challenges due to the fact that it accepts both **T** and **T1** as substrates, its inherent product promiscuity, poor expression in heterologous hosts and low catalytic activity (it has been shown to convert less than 10% of taxadiene to T-5α-ol). Most research efforts in the past 5 years were focused on T5αH, and more synthetic biology tools have been dedicated to T5αH than any other pathway enzyme to overcome this bottleneck on the second step of the pathway, thus, this review also has more comprehensive discussions on this enzyme.

To understand the origin of T5αH bottleneck, we present its history in heterologous expression in different hosts. Following successful cloning, functional expression and characterization of eight enzymes of the upper segment of the Taxol pathway, DeJong et al. (2006) sought to construct the first five sequential steps of the pathway leading to taxadien-5α-acetoxy-10β-ol in yeast (*Saccharomyces cerevisiae*). Expression and enzyme kinetics of T5αH in this construct was the lowest compared to other enzymes, leading to the first detection of a bottleneck at this first oxygenation step that restricted flux toward downstream steps. Two years later, an attempt to introduce TXS and T5αH in wild tobacco (*Nicotiana sylvestris*), targeting trichomes after knocking down production of cembratrien-diols similarly failed to produce the desired T-5α-ol, but instead led to production of a cyclic ether, 5(12)-oxa-3(11)-cyclotaxane (OCT) (Rontein et al., 2008) (summary of these metabolic engineering constructs are in Table 1). Intrigued by this observation, T5αH was expressed in yeast in the same study, and again OCT was observed. The bottleneck was again encountered in a carefully optimized *Escherichia coli* strain that was engineered via a multivariate modular metabolic engineering (MMME) approach that achieved a 15,000-fold increase in taxadiene production (titers of ∼1 g/L) but lost optimality and titers on introduction of T5αH (Ajikumar et al., 2010). Recent studies corroborated lack of selectivity and product promiscuity of T5αH *in vitro* and in several heterologous hosts like *E. coli*, *S. cerevisiae*, *Yarrowia lipolytica*, and *Nicotiana benthamiana*, showing a product profile dominated by OCT and its close isomer, iso-OCT, with T-5α-ol and several other monooxygenated diterpenes as minor compounds (Yadav, 2014; Biggs et al., 2016a, b; Edgar et al., 2016; Sagwan-Barkdoll and Anterola, 2017; Li et al., 2019). A number of optimization and engineering strategies that leveraged advances in synthetic biology have been applied to overcome the T5αH bottleneck as discussed in the sections below.

**TABLE 1:** Heterologous production of early taxane metabolites in different platforms.

Host	Details	Achievements	References
*E. coli*	Multivariate-modular metabolic engineering (MMME) approach to optimize the MEP pathway and GGPP-TXS as two operons under inducible promoters	Taxadiene accumulation to 1 g/L. Introduction of T5αH-CPR disrupted taxadiene balance and achieved ∼58 mg/L taxadiene-5α-ol and equal amounts of 5(12)-oxa-3(11)-cyclotaxane (OCT)	Ajikumar et al., 2010
*E. coli*	TXS and elaborately optimized T5αH and CYP450 reductase partner optimizations through N-terminal modifications, fusion linked chimera protein expression, and controlled promoter strength.	Oxygenated taxanes (570 mg/L) was achieved in a bioreactor	Biggs et al., 2016a
*E. coli*	Co-expression of dxs, idi, GGPPS, and TXS	Taxadiene (1.3 mg/L) in shake flask	Huang et al., 2001
*E. coli and S. cerevisiae*	A synthetic consortium was designed and genes for taxadiene-5α-10β-diol-acetate production were designed in 2 modules, taxadiene module in *E. coli* and acetylation and CYP450-oxygenation chemistry in *S. cerevisiae* for a stable co-culture fermentation using xylose as carbon source	33 mg/L oxygenated taxanes were achieved with TXS and T5αH, and adding T10βH and TAT achieved 1.0 mg/L of the target monoacylated, dioxygenated taxane	Zhou et al., 2015
*Bacillus subtilis*	Overexpression of all MEP pathway genes (*dxs*, *ispD*, *ispF*, *ispH*, *ispC*, *ispE*, *ispG*) together with *ispA*, GGPPS and TXS	Taxadiene accumulated to 1.98 mg/L/OD_600_ (17.8 mg/L) in shake flask	Abdallah et al., 2019
*S. cerevisiae*	Multi-step pathway construction of 5 taxoid biosynthetic genes (GGPPS, TXS, T5αH, TAT, and T10βH) to attempt taxadiene-5α-acetoxy-10β-ol production.	All 5 recombinant proteins were successfully expressed and had measurable activity. Only Taxadiene (1 mg/L) and trace amounts of taxadiene-5α-ol (∼25 μg/L) was detected. No advanced metabolites were detected	DeJong et al., 2006
*S. cerevisiae*	Heterologous expression of a truncated 3-hydroxyl-3-methylglutaryl-CoA reductase (tHMGR), a mutant regulatory protein, UPC2-1, GGPPS from *Sulfolobus acidocaldarius* and TXS	Taxadiene (8.7 mg/L) and geranylgeraniol (33 mg/L) accumulated in shake flasks after 48 h fermentations	Engels et al., 2008
*S. cerevisiae*	Heterologous TXS and GGPPS (from *Taxus cuspidate × Taxus baccata*) and overexpression of erg20 and tHMGR	Taxadiene accumulated to 72.8 mg/L	Ding et al., 2014
*S. cerevisiae*	A CRISPR/Cas 9 toolkit was tested on TXS expression optimization in yeast. 10 protein tags and 5 promoters of different strengths were tested. Fusion of TXS to MBP under the strong GAL1 promoter achieved highest titer	Taxadiene titer of 20 mg/L was achieved	Apel et al., 2017
*S. cerevisiae*	TXS-ERG20 fusion protein was constructed with MBP tag for improved solubility, together with promoter strength and growth temperature optimization	High taxadiene titer in yeast of 129 mg/L was achieved in a bioreactor	Nowrouzi et al., 2020
*A. thaliana*	Chimeric TXS cDNA constitutively expressed in *A. thaliana*	Taxadiene (∼20 ng/g DW) in seedlings and leaves, but however observed stunted growth and reduced photosynthetic pigments. Induction with the synthetic glucocorticoid (dexamethasone) improved yields to 600 ng/g DW	Besumbes et al., 2004
Tobacco (*Nicotiana sylvestris*)	TSX and T5αH were stably expressed in tobacco trichome cells	Taxadiene (no reported yield) was detected while expected taxadiene-5α-ol was not detected in leaf extracts. Instead, only OCT was detected. Yeast microsomes also produced OCT only	Rontein et al., 2008
Tomato fruits	TXS was stably transformed into a yellow-fruited tomato line which lacks a functional phytoene synthase	160 mg/kg from freeze-dried tomatoes	Kovacs et al., 2007
Tobacco (*Nicotiana benthamiana*)	TXS, truncated T5αH and cytochrome P450 reductase were inserted into the chloroplast compartment and precursor pathway was overexpressed	Taxadiene—56.6 μg/g FW and Taxadiene-5α-ol was detected for the first time in a heterologous plant platform at 1.3 μg/g fresh weight	Li et al., 2019
Ginseng (*Panax ginseng*) roots	Stable transformation of TXS from *Taxus brevifolia* into ginseng roots	TXS-transgenic ginseng accumulated 9.1 μg/g DW. Methyl jasmonate treatment improved yields to 14.6–15.9 μg/g DW	Cha et al., 2012
*Physcomitrella patens* (moss)	Stable constitutive expression of TXS using a ubiquitin promoter	Taxadiene accumulated to 0.05% FW of plant tissue. No adverse effects on growth were noted	Anterola et al., 2009
*Alternaria alternata* (endophytic fungus)	Co-overexpression of isopentenyl diphosphate (*idi*), truncated 3-hydroxy-3-methylglutaryl-CoA reductase (tHMG1) and TXS under different promoter strengths	Detection of 61.9 μg/L taxadiene after 14 days of fermentation	Bian et al., 2017

### Poor Pathway Flux and the Branched Nature of the Pathway

Despite that much of the Taxol pathway enzymes and genes have been identified, success in engineering at least the known segments of the pathway in heterologous systems have been mainly hampered by inherent poor enzyme catalysis, protein interdependency issues and product promiscuity of key enzymes that lead to a highly branched pathway. Carbon flux is channeled toward off-target products by the promiscuous enzymes, presenting a formidable challenge to synthetic biologists that cannot be solved by simple redirection of IPP and DMAPP precursors. The first enzyme, TXS is now known to have a broad substrate profile including verticillenes and reports of cembrene A, in addition to **T** and **T1**, as discussed below. The T5αH- catalyzed second step splits **T** into many other products, dominated by OCT and its isomer, iso-OCT, branching the pathway into many directions. Several other downstream enzymes also accept different substrates and have broad product profiles, which present an emerging picture of a highly branched pathway. This lack of linearity is at the core of the problems with the pathway that has not only slowed metabolic engineering advances, but has even prevented pathway elucidation and identification of key downstream intermediates. Not surprisingly, recent work has focused on improving the catalytic efficiency and selectivity of pathway enzymes through experimental methods supported by computational tools to improve our mechanistic understanding of catalysis, as covered in the next sections.

### Lack of Knowledge on the Regulatory Mechanisms and *in planta* Transport Mechanisms

Not much is known on the regulation of the Taxol pathway at transcription, translation and post-translational levels. Elicitors like methyl jasmonate (MeJA) have been used for a relatively long time in improving secondary metabolism in *Taxus* cell suspension cultures, but the mechanisms through which MeJA activates Taxol biosynthesis have not been elucidated in detail. Knowledge of the regulatory mechanisms, identification of key transcription factors and any feedback loops in the pathway is critical in informing metabolic engineering efforts. Likewise, there are also many gaps in our understanding of the transport mechanisms of taxane intermediates in plant cells. This wealth of information will be very vital in designing a biosynthetic route to Taxol especially in plant cells.

## Advances in Metabolic Engineering, Protein Engineering and Mechanistic Insights on Taxol Related Ezymes

Synthetic biology tools have been widely applied in advancing Taxol biosynthesis research from gene discovery to pathway designs and construction in heterologous hosts, resulting in successes in detection and accumulation of taxane intermediates. Recent metabolic engineering strategies and achievements in different hosts are summarized in Table 1, together with approaches used in previous years, for comparison. Computational tools are an enabling technology that has also been at the forefront in unraveling mechanistic insights of key enzymes to guide protein engineering strategies for use in metabolic engineering constructs, thus we discuss these advances together in this section.

### Quantum Mechanics/Molecular Mechanics (QM/MM) and Computational Modeling of a Catalytically Active TXS to Enable Enzyme Engineering

The class 1 terpene cyclase taxadiene synthase (TXS) is the first and rate-limiting enzyme of the Taxol biosynthesis pathway. It accepts the acyclic C20 diterpenoid precursor, (*E,E,E*)-geranylgeranyl diphosphate (GGPP) and cyclizes it to the endocyclic diterpene olefin taxa-4(5),11(12)-diene (taxadiene, **T**) and four other minor products: taxa-4(20), 11(12)-diene (isotaxadiene, **T1**), verticillia-3(4),7(8),12(13)-triene (**V**), verticillia- 4(20),7(8),11(12)-triene (**V1**) and verticillia-3(4),7(8),11(12)-triene (**V2**) (Koepp et al., 1995; Lin et al., 1996; Schrepfer et al., 2016; Li et al., 2019) and also the isomer taxa-3(4),11(12)-diene (Williams et al., 2000b; Li et al., 2019). Though the X-ray crystal structure of TXS was solved almost a decade ago (Köksal et al., 2011), it lacks N-terminal residues and is in an open, catalytically inactive form, which does not provide much clues relevant to the mechanism and intricate architecture of the active form. In addition, the reported structure is bound to the fluorinated substrate, 2-fluoro-geranylgeranyl diphosphate (2-F-GGPP) in a non-productive orientation (Hong and Tantillo, 2011; Schrepfer et al., 2016).

Consequently, labeling and computational tools have been used to get insights on the energetics of proton transfer and carbocation formation in the TXS active site. The generally agreed mechanism follows TXS-catalyzed cleavage of the pyrophosphate moiety (PPi) from GGPP to form charged carbocations followed by subsequent cyclizations and proton transfer, leading to the mixed product profile stated above (Williams et al., 2000a; Köksal et al., 2011). Using QM calculations, an indirect, two-step protein transfer sequence was proposed (Gutta and Tantillo, 2007; Hong and Tantillo, 2011). However, these gas phase calculations assumed a passive role of the TXS protein and placed much emphasis on substrate reactivity. To improve on this prior computational work and identify the role of the deprotonating bases in the TXS active site, more recent work focused on building closed, active models of the protein. Two groups have so far successfully used homology modeling and loop modeling based on the closed structure of bornyl diphosphate synthases (BPPS) as a template to model the missing N-terminal residues and build catalytically active models that can be used in docking the productive substrate, GGPP (Schrepfer et al., 2016; Freud et al., 2017). Such structural model based molecular mechanics calculations have the potential to inform enzyme engineering strategies to generate pathway enzyme variants with improved catalytic efficiency, less product promiscuity or tailored product profile to enable selective attenuation of the carbocation products. For example, closed TXS model construction and molecular mechanics by Schrepfer et al. (2016) led to identification of a conserved amino acid network responsible for an extended hydrogen (H) bonding (water- and amino-acid mediated) involving the PPi and R754, R768, Y835, R580, and N-terminal Y89 in the closed TXS-GGPP complex. Targeted mutagenesis of these five residues lead to loss of activity (Table 2), and the W753 residue was identified as a key deprotonating base for the Cation B (cembren-15-yl cation) that resulted in cembrene A formation when mutated to a histidine (W753H) or in a double mutant (W753H/C830A) (Table 2; Ansbacher et al., 2018; Schrepfer et al., 2016). In fact, the biotechnological application of these TXS-derived mutants was demonstrated, with W753H yielding 8 mg/L cembrene A and V584M yielding 11 mg/L verticilla-3,7,12(13)-triene in 30 L batch fermentations (Schrepfer et al., 2016).

**TABLE 2:** Mutagenesis of enzymes of the Taxol pathway to manipulate catalytic fitness, activity and product distribution profile.

Target	Mutation	% Activity	Taxadiene (T)	Iso-taxadiene (T1)	Verticillenes	Cembrene A	References
**TXS**							
TXS	Wild type	100	93.2	4.7	2.1	N.D	Schrepfer et al., 2016
Y89	Y89A/E/F	Lost activity*					
R580	R580A/E/H	Lost activity					
R754	R754A/E/H	Lost activity					
R768	R768A/E/H	Lost activity					
Y835	Y835A/F/W	Lost activity					
V584	V584K/L/M	89–92	14–30	0.6	70–85.6		
	V584N/S/P/R	Lost activity					
S587	S587D/Y/K/L/G	Lost activity					
	S587A	21.8	8.9	N.D	32.2	58.9	
Y609	Y609G	N.A	N.D	N.D	100	N.A	Edgar et al., 2017
V610	V610H/S/F/A	Lost Activity					Schrepfer et al., 2016
S713	S713T	97.4	92.7	5.1	2	N.D	Schrepfer et al., 2016
	S713A/L	Lost activity					Schrepfer et al., 2016
V714	V714A/I	8.7–10.4	94	5.7–5.9	N.D	N.D	
	V714T/G/P	Lost activity					
G715	G715A/S	Lost Activity					
S713	S713T	97.4	N.D	N.D	N.D	100	
	S713A/L	Lost activity					
W753	W753H	51.3	N.D	N.D	N.D	100	Schrepfer et al., 2016; Ansbacher et al., 2018
	W753H/C830A	48.5	N.D	N.D	N.D	100	Schrepfer et al., 2016
	W753A/C/E/L/V	Lost activity					
C830	C830A	88.5	93.1	4.8	2.1	N.D	
	C830S	92.4	79.3	7.3	13.4	N.D	
F834	F834A/G	25.6–28.3	38–87	6.1–6.5			
Y835	Y835F	3.5	100	N.D	N.D	N.D	
	Y835A/W	Lost activity					
Y841	Y841F	41.3	N.D	N.D	43.6	56.4	
	Y841A/T	Lost activity					
Y688***^¶^***	Y688L	2.4-fold increase in **T1**, and corresponding increase in T-5α-ol					Edgar et al., 2017
**T5αH**							
T380	T380S	Produced a dihydroxylated product—5(12)-oxa-3(11)-cyclo-taxan-10-ol					Edgar et al., 2017
K131	K131R	Improved turnover but lost selectivity compared to wild type					Yadav, 2014
V374	V374L	Improved selectivity at the expense of turnover					
S302	S302A	Lost activity and no change in fold-change					
**DBAT**							
G38	G38R	2.15-fold increase in baccatin III					Li et al., 2017
H162	H162A	Lost activity					Li et al., 2017
	H162A/R63H	3-fold increase in catalytic activity compared to wild type					You et al., 2018
R363	R363A	Lost activity					Li et al., 2017
	R363H	26-fold increase in catalytic activity compared to wild type					You et al., 2018
G361	G361A	Lost activity					Li et al., 2017
I164	I164A	Lost activity					Li et al., 2017
D166	D166H	15-fold increase in catalytic activity compared to wild type					You et al., 2018
	D166H/R363H	60-fold increase in catalytic activity compared to wild type					You et al., 2018
I43/D390	I43S/D380R	3.3-fold increase in catalytic efficiency using vinyl acetate and 3-fold using acetyl CoA					Lin et al., 2018

*Enzyme activity in comparison to the wild type TXS. Activity of 0–2% was qualified as “lost activity”. N.D., Not detectable; N.A., Not assayed. **^¶^** These mutants led to corresponding increases in taxadiene-5α-ol after introduction of T5αH-CPR.

Using the TXS model constructed by Freud et al. (2017), the crucial role of W753 and the analogous Y841 in controlling highly charged carbocations in the hydrophobic TXS active site were highlighted in simulations by Ansbacher et al. (2018). However, recent molecular dynamics (MD) and QM/MM calculations place more emphasis on the role of the reactive carbocations, the retained PPi and active site water molecules in controlling carbocation cascades and product distribution, rather than the TXS residues (Escorcia et al., 2018; van Rijn et al., 2019). The calculations corroborate the critical role of R580 in deprotonation of carbocation C, but suggest this is through water-assisted deprotonation via one or two water bridges, rather than direct interaction with PPi and closure of the active site (as a part of the RXR motif of terpene synthases) as suggested by Freud et al. (2017). Another interesting take from these computational and experimental data sets was the manipulation of the taxadiene and iso-taxadiene product percentage, as this has been shown to be important in determining the selectivity of the subsequent CYP450-catalyzed step as described below. C830S yielded the highest iso-taxadiene without much reduction in overall activity (Table 2), and such a mutant can be leveraged for improving the selectivity of T5αH as demonstrated by Edgar et al. (2017) (described below).

### Strategies Toward Overcoming the T5αH Bottleneck

#### Optimization of Oxygenation Chemistry

**FIGURE 2:**
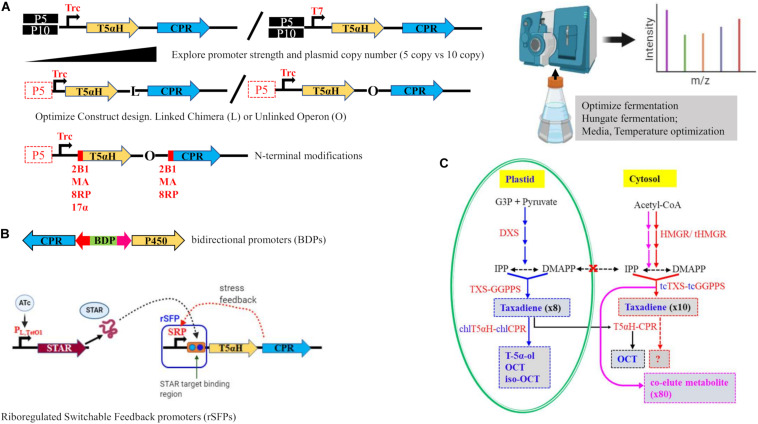
Tools and strategies used to overcome the T5αH bottleneck. **(A)** Optimization of the P450 module in a previously optimized *E. coli* strain with chromosomally integrated MEP and TG modules was achieved using several approaches (see Biggs et al., 2016a). Top panel—A high copy number plasmid (P10) was compared to a low copy number (P5), and expression strength was explored using a strong T7 promoter and a weak (Trc) promoter; Middle panel—Using a low copy P5 plasmid and weak Trc promoter, interactions of the P450 and its reductase partner CPR were explored using a linked chimera construct (L) or an unlinked operon design (O); Lower panel—N-terminal modifications were tested on truncated versions of T5αH and CPR to enhance solubility using three leader peptides—2B1, MA an 8RP; 17α was also used in another study (Rouck et al., 2017). Right panel—Further optimizations including targeted proteomics and optimization of media and fermentation conditions led to highest titers of oxygenated taxanes. **(B)** Targeting promoters for dynamic regulation of T5αH catalysis. Top—Bidirectional promoters (BDPs) that differ in inducibility and strength can be used for fine-tuning and timing of expression of proteins in the Taxol pathway. Vogl et al. (2018) tested this concept and achieved a 50x-improvement in taxadiene titers. Lower—Glasscock et al. (2019) introduced riboregulated Switchable feedback promoter (rSFP) that uses a natural stress-response promoter (SRP) in combination with a second plasmid containing a small transcription activating RNA (STAR) that can be induced by an inducer like anhydrotetracycline (ATc) to create an external ON/OFF gate that achieved a 2.4-fold increase in oxygenated taxanes compared to Biggs et al. (2016a). **(C)** Compartmentalized engineering in plant tissue (*N. benthamiana*) targeting chloroplasts (Li et al., 2019) and cytosol (De La Peña and Sattely, 2020) was conducted. Plastid—chloroplastic targeting (blue arrows) resulted in production of T-5α-ol for the first time, while cytosolic targeting (red arrows) resulted in 10-fold increase in taxadiene. Purple arrows—upregulation of HMGR without cytosol-targeted tcTXS-tcGGPPS led to 80x increase in an unidentified compound that co-eluted with taxadiene. Black arrows—introduction of DXS and TXS-GGPPS without chloroplastic targeted chlT5αH-chlCPR results in detection of OCT only. Red dotted arrow, cytosolic engineering of taxadiene with further introduction of T5αH-CPR has not yet been attempted.

Realizing the magnitude of the bottleneck posed by T5αH, Biggs et al. (2016a) carried out an extensive study to optimize P450 chemistry in *E. coli* as a proof of concept and achieved a fivefold increase in oxygenated taxanes, reaching the highest oxygenated diterpene titer to date (∼570 mg/L). The work built from the MMME breakthrough (Ajikumar et al., 2010) and aimed to optimize downstream P450 chemistry through chromosomal integration of the upstream pathways and use of different strategies to optimize T5αH and cytochrome P450 reductase (CPR) partner interactions. A protein interdependency of the oxidative module and the upstream modules was uncovered through targeted proteomics, and was determined as a key obstacle of T5αH expression and to be responsible for reduction of upstream metabolites on introduction of T5αH. Strategies used to uncouple this interdependency and optimize the pathway proteins were varying promoter strength, N-terminal modifications, gene copy number optimization and optimization of CPR interactions (Figure 2A). Construction of the T5αH-CPR module as an operon resulted in higher titers compared to a linked, chimera construct; and a weaker Trc promoter demonstrated overall higher yields compared to a stronger T7 promoter. These results suggested the need for a lowered CPR expression as evidenced by the benefits of an operon construction and Trc promoter. To address the solubility issue that is common with P450 enzymes, truncation of the hydrophobic, membrane-targeting N-termini of both T5αH and CPR was undertaken, and three leader peptides of different solubilities (8RP, MA, and 2B1) were attached for N-terminal modifications. Despite the significant improvement in solubility of the expressed respective proteins that was noted, there were no benefits of these modifications on T5αH performance, in fact, increased hydrophilicity (2B1-T5αH/2B1-CPR) resulted in accumulation of taxadiene that was not converted to oxidized taxanes. Ultimately, this work developed a strain with a chromosomally integrated MMME module and T5aH-CPR in an operon construct in a low copy plasmid under a weak Trc promoter as the most optimal that achieved ∼570 mg/L oxygenated taxanes in a benchtop bioreactor.

As demonstrated by Biggs et al. (2016a), optimizing promoter strength to strike a balanced expression of pathway enzymes or achieving dynamic expression is a very powerful tool in metabolic engineering and synthetic biology. In the context of the Taxol pathway, this tool was recently exploited to finely tune expression of TXS and GGPPS using bidirectional promoters (BDPs) in the yeast *Pichia pastoris* (Vogl et al., 2018). BDPs allow not only differential expression of genes utilizing differences in promoter strength, but can be used to explore constitutive and inducible promoters, and for timing where one gene needs to be expressed after another (Figure 2B). When GGPPS was expressed under a constitutive promoter, no taxadiene was detected, but when GGPPS was under a depressed promoter and TXS under a different promoter, taxadiene titers increased by nearly 60-fold (Vogl et al., 2018). Though this strategy was not extended to T5αH, the same study already provided a promising example with a human P450 and its CPR partner that improved by fivefold under an optimal BDP. Another example of the versatility of manipulating promoters for dynamic pathway expression to overcome the T5αH bottleneck is a recent report using riboregulated switchable feedback promoters (rSFPs) (Glasscock et al., 2019). These novel rSFPs are created through using a natural, stress-response promoter in conjunction with a target sequence that is inserted between the promoter and the gene. A separate *trans*-acting regulator, called small transcription activating RNA (STAR) is introduced in a second plasmid to bind and activate the rSFP, creating a gated ON/OFF switch for the downstream gene (Figure 2B). Working with the best optimized strain from Biggs et al. (2016a), this rSFP tool was used to screen membrane envelope stress-response promoter library with the goal to create a promoter that is responsive to the stress caused by introduction of T5αH/CPR to the upper pathways. Optimization of the timing and expression magnitude of T5αH/CPR with rSFP in this way resulted in a notable improvement of 2.4-fold (25.4 mg/L) in oxygenated taxanes and 3.6-fold (39.0 mg/L) increase in total taxanes (Glasscock et al., 2019).

CRSIPR/Cas9 technology is a fast and precise enabling tool that is increasingly being used in synthetic biology applications. This technology was applied to build a cloning—free screening toolkit for promoter strength and solubility tag optimization in *S. cerevisiae* (Apel et al., 2017) to enable quick exploration of different constructs. As a proof of concept, the toolkit was applied to build an expression context library for TXS to explore localization tags, solubility tags and promoter strength. The fast toolkit identified a solubility problem with TXS in yeast as the major cause of poor catalysis, and identified the best optimized construction of TXS with MBP solubility tag and a strong GAL1 promoter that increased titers 25-fold compared to an optimized strain.

#### T5αH Protein Engineering

**FIGURE 3:**
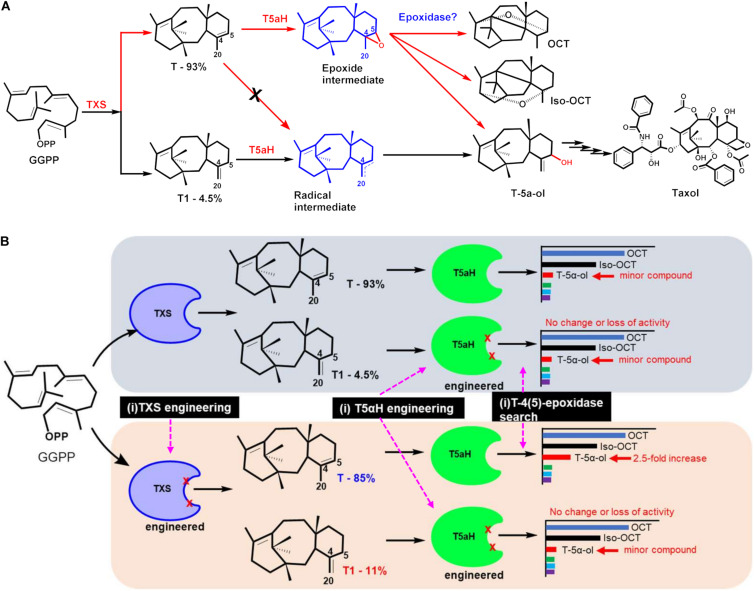
Improving T5αH catalysis through mechanism—informed protein engineering strategies (based on Edgar et al., 2016, 2017). **(A)** A novel mechanism of T5αH catalysis was proposed, via an unstable epoxidase intermediate. Under this proposal, the mechanism of T5αH catalysis is substrate controlled. Taxadiene **(T)** undergoes catalysis through the proposed epoxide (red arrows), leading to several products like OCT, T-5α-ol and iso-OCT, while iso-taxadiene **(T1)** follows the radical rebound mechanism (black arrows) that exclusively leads to the desired T-5α-ol product and ultimately to Taxol. **(B)** Exploring engineering targets to improve T5αH catalytic specificity toward T-5α-ol. Three strategies: (i) TXS engineering to improve iso-taxadiene yield, (ii) T5αH engineering to improve catalytic efficiency and specificity and (iii) mining for an epoxide that could convert the epoxidase to T-5α-ol. Engineered TXS with improved T1 percentage improved T-5α-ol yield 2.5-fold.

While all the above tools are promising for optimizing T5αH expression, it is important to note that the improvements are in titers of “oxygenated taxanes”, a term describing a mixture of different mono- and doubly oxygenated taxanes monitored at m/z 288 and m/z 304 in the GC-MS chromatograms. The only product of T5αH that has been shown to lead to Taxol is T-5α-ol, thus, though increases in total oxygenated taxanes are promising, it is highly desired to address product promiscuity of the enzyme, and devise ways to improve only the desired product (i.e., T-5α-ol). Protein engineering is an effective tool that has already been harnessed to address the T5αH bottleneck, and to improve other enzymes of the Taxol pathway (Figure 3). Notwithstanding major advancements in directed evolution as a subfield that recently won a Nobel price, the major challenges with leveraging this tool in engineering T5αH and other P450s of the Taxol pathway is the dearth of mechanistic knowledge of catalysis and the lack of high throughput screening assays to quickly screen the large number of generated mutants. Assuming that the experimentally observed product promiscuity of T5αH was due to competing regiospecific proton abstraction by the oxyferryl species of the P450 enzyme on taxadiene as proposed previously (Hefner et al., 1996; Jennewein et al., 2004), a computational method was developed to guide mutagenesis and improve catalytic efficiency and selectivity (Yadav, 2014). A total of 53 mutants were designed targeting amino acids residues around the active site of the enzyme, basing on an energy-minimized homology model of T5αH that was developed using six P450 structures as template. Assessment of the mutant library identified six variants (5 single and 1 triple mutant) that improved both in turnover and T-5α-ol selectivity, though the identities of the mutants were not clearly stated and the mechanistic basis of the improvements was not discussed. Three mutants from the study were however used to infer mechanistic basis on the observed changes; S302A, K131R, and V374L (Table 2). Product promiscuity was slightly improved in the identified mutants, but T-5α-ol remained a minor product while OCT and iso-OCT dominated the product profile.

A different T5αH mechanism involving an epoxide intermediate, as opposed to an exclusive radical intermediate, was suggested by the Stephanopoulos group (Edgar et al., 2016). Under this proposal, the two main products of TXS (**T** and **T1**) undergo catalysis through different transition states en-route to T-5α-ol; **T1** follows the radical-rebound mechanism (abstraction at the C20- position), while **T** follows epoxidation route to an unstable epoxide intermediate that decomposes non-selectively to several products like OCT, iso-OCT, and T-5α-ol (Figure 3A). The proposal was supported by several lines of evidence, and it was experimentally determined that incubating taxadiene with TXS leads to several products, but incubating with iso-taxadiene produced a single peak of T-5α-H. Another independent chemical synthesis study provided direct evidence that indeed **T** can be epoxidized in a regio- and diastereoselective manner to yield the intermediate taxadiene-4(5)-epoxide that can be further rearranged into T-5α-ol, OCT, and iso-OCT (Barton et al., 2016). These observations are significant, and support that T5αH catalysis is substrate-driven, and that the broad product profile is a result of non-selective epoxide degradation rather than T5αH non-selectivity as previously assumed. Based on this alternative mechanistic proposal, three targets can be manipulated for enhancing selectivity of T5αH hydroxylation; (i) manipulating TXS selectivity to enhance **T1** in the product profile, (ii) engineering T5αH to improve turnover and (iii) searching for an epoxidase enzyme to improve selectivity of the hypothesized epoxidase intermediate (Figure 3B). All three approaches were conducted and TXS engineering to enhance **T1** titers resulted in variants that displayed 2.4-fold improvement in **T1** leading to a 2.4-fold improvement in T-5α-ol titers in *E. coli* after reconstituting T5αH-CPR (Edgar et al., 2017). A total of 14 residues consisting of polar residues near the TXS active site, residues closer to PPi, and two residues in the N-terminus that caps the active site were investigated by saturation mutagenesis, and the most successful mutant was Y688L. In fact, in addition to the critical PPi assisted abstraction, docking simulations also identified Y688 as a critical residue for abstraction of the hydrogen on C-5 position. Mutation of N-terminal residues led to a loss of activity, a result that was in agreement with Schrepfer et al. (2016). Mutagenesis of the P450 enzyme and mining of a novel T-4(5)-epoxidase were not successful, but with the increasing amount of *Taxus* transcriptome datasets and the substrate synthesis methodology reported by Barton and colleagues, further querying, *in vitro* screening and activity-guided fractionation are promising methods in searching for this enzyme. In addition to T-5α-ol-enhancing mutations, other TXS mutants were identified that yield verticillines (Q609G), or other unidentified compounds, most likely cembrene A (Table 2).

#### Semi-Rational Designing of DBAT

Besides the first two enzymes of the Taxol pathway, other downstream enzymes have also been targeted for engineering enhanced catalysis. For example, DBAT was engineered with the aid of a computational semi-rational strategy, leading to a double mutant (I43S/D390R) that not only improved catalytic efficiency but could more efficiently utilize vinyl acetate, a cheaper alternative acyl donor compared to acetyl CoA (Lin et al., 2018). Compared to the wild type DBAT, the double mutant improved catalytic efficiency by 3.3-fold when using vinyl acetate, and 2.99-fold when using acetyl CoA (Table 2). Employing a similar computationally guided semi-rational design, Li et al. (2017) employed structure modeling, molecular docking, alanine scanning and saturation mutagenesis to engineer DBAT for acetylation of the C10 position of 10-deacetyltaxol (DT) with the aim to utilize the C7-gylcosylated Taxol analog (7-β-xylosyl-10-deacetyltaxol) for Taxol biosynthesis. The double mutant engineered in the study (G38R/F301V) demonstrated 6-fold improvement in catalytic efficiency (acetylation of DT to Taxol) compared to wild type DBAT, and in addition, a mutant (G38R) was identified with 2.15-fold improved catalytic efficiency in converting the native substate (10-deacetyl baccatin) to baccatin III. Molecular docking also revealed the critical catalytic role of His162 in DBAT catalysis, and alanine scanning identified 4 residues that led to a complete loss of activity when mutated to alanine (H162A, R363A, G361A, and I164A). Two of these residues (H162 and R363) and an additional active site residue (D166) were further investigated by another group by a similar computational strategy supported by site directed mutagenesis (You et al., 2018). Postulating the benefits of histidine residues in the DBAT catalytic pocket, these residues were mutated to histidine, leading to D166H, R363H, H162A/R363H, and D166H/R363H that demonstrated superior catalytic activities (15-, 26-, 3-, and 60-times improvements compared to wild type DBAT, respectively) (Table 2).

Computational tools are increasingly becoming useful in gaining mechanistic insights on the catalysis cycles of Taxol pathway enzymes. The above examples highlight how versatile and powerful the tool was leveraged for several proteins. Cytochrome P450s are very sensitive to mutations, thus computational and phylogenetically guided mutagenesis studies of T5αH have so far not yet identified significantly improved mutants for solving this bottleneck. Nevertheless, given the lack of a X-ray crystal structure of T5αH or any CYP450 of the Taxol pathway, homology modeling and molecular docking have thus far provided three models (Edgar et al., 2017; Rouck et al., 2017; Yadav, 2014) that can guide semi-rational design strategies. Several residues were identified in the resulting docking conformations that are potentially involved in stabilizing the hydrophobic taxadiene, reaction intermediates and the heme that could shed light on the mechanism of catalysis.

#### Metabolic Engineering in Plant Hosts

Plant hosts are expected to offer a more favorable environment for the challenging functional expression of plant CYP540 that currently frustrates engineering of the pathway in microbial hosts. Taxol pathway genes are located in different organelles in the plant cell (cytosol, endoplasmic reticulum membranes, and chloroplasts) but the nature of the exchange of intermediates among these different locations is not known. We considered inter-organellar transport of taxane intermediates to be the major barrier blocking access of endoplasmic reticulum-localized CYP450s to their diterpenoid substrate produced in the plastid in plant cells and used a compartmentalized engineering strategy that led to production of T-5α-ol in a heterologous plant host for the first time (Li et al., 2019; Figure 2C). A very recent report similarly targeted TXS and GGPPS in the cytosol and overexpressed the mevalonate pathway rate limiting enzyme 3-hydroxy-3-methylglutaryl-CoA reductase (HMGR) and observed a 10-fold improvement in taxadiene yields in *N. benthamiana* leaves (De La Peña and Sattely, 2020). Given the robustness of the mevalonate pathway compared to the MEP pathway, cytosolic targeting could potentially open new avenues for engineering taxanes in plant cells. It remains to be tested if this cytosolic compartmentalization strategy could be leveraged for engineering production of oxygenated taxanes.

## Novel Candidate Genes and Insights From Recent Transcriptome Data

Advances in sequencing technology in the last decade opened avenues for gene discovery and pathway elucidation of Taxol biosynthesis. Almost all novel genes identified in the early days were discovered through leveraging homology-based cloning, random sequencing of cDNA libraries from MeJA-elicited *Taxus* cell cultures, differential display of mRNA-reverse transcription-PCR, screening of EST libraries and use of available substrates and surrogate substrates (see reviews by Walker and Croteau, 2001; Kaspera et al., 2006). The advent of high throughput RNA sequencing technologies presented a very powerful tool that enabled transcriptome and genome sequencing, generating a huge amount of data that often is challenging to analyze and infer meaningful biological relevance. To date, powerful sequencing technologies using next-generation sequencing (NGS) and third-generation sequencing (TGS) platforms have been applied in transcriptomic studies of *Taxus* tissues and cell suspension cultures. Recent years have witnessed an increased interest in sequencing Taxol-related transcriptomes, revealing unprecedented insights into regulatory mechanisms of the pathway, unraveling mechanistic links to plant hormone signal transduction pathways and providing several lead candidates for the missing pathway genes—with some that have already been confirmed functional.

A transcriptome study was conducted with MeJA-elicited *T. baccata* suspension cells using high throughput complementary DNA-amplified fragment length polymorphism (cDNA-AFLP) that provided a total of 15 candidate transcripts identified as potential lead candidate genes encoding the six remaining enzymes (PCL, T1βH, T9O, C4-C20 epoxidase, T2′αH, and oxomutase) of the Taxol pathway. Functional characterization of these candidates led to the isolation of PCL (Ramírez-Estrada et al., 2016). A combined transcriptomic assembly of *Taxus chinensis* cultured cells and *in silico* mining of publicly available transcriptome data sets covered a comprehensive list of CYP450 genes, creating a valuable resource for searching the missing enzymes and for finding alternative P450s for bottleneck enzymes like T5αH (Liao et al., 2017). A total of 118 full length and 175 partial length *T. chinensis* P450s were identified, including the five known P450s of the pathway (CYP725A1—T10βH; CYP725A2—T13αH; CYP725A4—T5αH, CYP725A5—T7βH, CYP725A6—T2αH) and six novel CYP725A subfamily genes (CYP725A9, CYP725A11, CYP725A16, CYP725A20, CYP725A22, CYP725A23). The same three sets of publicly available transcriptome datasets from *T. chinensis* cultured cells were mined for WRKY transcription factors leading to identification of 61 transcripts of TcWRKY of which six selected genes were all upregulated by MeJA (Zhang et al., 2018a).

Other recent Taxol-related transcriptomes covered a taxol-producing endophytic fungi *Cladosporium cladosporioides* MD2 (Miao et al., 2018), profiling of time-series reprogramming of *Taxus x media* genes following MeJA treatment (Mao et al., 2018), comparison of wild type *T. yunnanensis* with a high Taxol and 10-DAB—yielding new cultivar (He et al., 2018), comparative transcriptomes of *T. media*, *T. marei*, and *T. cuspidata* that differ in Taxol content (Zhou et al., 2019) and Iso-Seq of *T. cuspidata* tissues (Kuang et al., 2019). The Iso-Seq transcriptome identified nine CYP450s and seven acyl transferases (ACTs) as possible lead candidates for Taxol biosynthesis. The utility of transcriptome data sets in novel gene discovery, unraveling of biosynthetic and regulatory mechanisms was demonstrated in numerous examples discussed above. An in-depth computational and experimental analysis of the cDNA-AFLP dataset previously analyzed by Ramírez-Estrada et al. (2016) was recently conducted by the same group, leading to identification of transcript TB506 as a putative Taxane 2′a hydroxylase (T2′αH) (Sanchez-Muñoz et al., 2020). Molecular docking was conducted to confirm binding of such a huge substrate as 3′N-dehydroxydebenzoyltaxol and possible conformations were achieved. Functional expression and characterization of T2′αH activity was confirmed in *Pisum sativum* protoplasts, opening a biotechnological route to Taxol from its available intermediates 10-DAB and BIII.

In another recent study relevant to biotechnological production of Taxol, a library of 17 acyl CoA transferases (ACTs) was mined from three publicly available RNA-Seq data sets (from MeJA-elicited *T. media* suspension cell cultures) and screened for activation of different organic acids for N-substitution of 3-phenylisoserine side chain of taxoids (Srividya et al., 2020). In addition to identification of a candidate ACT with high specificity for generating CoA ester of benzoic acid (leading to Taxol formation) the study identified and functionally characterized several ACTs responsible for inserting different groups on this position, leading to several observed taxoids like Taxol B (insertion of a hexanoic acid), Taxol C (tiglic acid) and Taxol D (butyric acid). An ACT clone with 99% identity to the one described above (Ramírez-Estrada et al., 2016) was isolated, but activity screening of the clone, and all other candidates by Srividya et al. (2020) did not yield a positive hit.

Biochemical assays have been used to confirm several regulatory mechanisms inferred from deep sequencing studies. Using a GUS reporter assay with promoters of seven pathway genes from *Taxus cuspidata* cells, three basic helix-loop-helix (bHLH) transcription factors (TcMYC1, TcMYC2, and TcMYC4) were identified as negative regulators of MeJA-induced Taxol biosynthesis through their interaction with E-boxes in the promoters of Taxol pathway genes (Lenka et al., 2015). A more recent study, however, used GUS reporter assays in combination with yeast-one-hybrid, yeast-two-hybrid and *in vitro* assays and revealed TcMYC2a as a positive regulator of TXS in JA signaling (Zhang et al., 2018b). It relays its positive signal through binding JAZ proteins, and interacting with promoters of ERF15, ERF12, and TXS through the T/G-box, G-box, and E-box in their promoters.

## Perspectives and Concluding Remarks

Most genes of the Taxol pathway were isolated and their encoded proteins were functionally characterized is different systems. However, successes in functional expression of individual genes were not replicated when sequential genes were constructed into a pathway to synthesize intermediates. The most successful heterologous production of a taxane intermediate was 1 g/L achieved 10 years ago through an MMME approach, but introduction of T5αH that catalyzes the second step to the strain led to a dramatic loss of both optimality and titers. The past 5 years witnessed a significant increase in application of synthetic biology tools together with the emerging enabling technologies for gene assemblies in enzyme discovery and metabolic engineering of taxane intermediates. Several synthetic biology tools have been leveraged to optimize T5αH expression and activity, including truncations, promoter optimization, CPR optimization and compartmentalized engineering in plant organelles and use of riboregulated switchable feedback promoters (rSFPs). Computational and experimental approaches were used to improve our understanding of the catalytic mechanism of TXS and T5αH, shedding more light on the transition states governing the observed product profile. Models of the closed TXS and those of T5αH and DBAT were also built that provided intricate details of the active site architecture to guide semi-rational protein engineering strategies to improve catalytic activity and alter product profile.

As highlighted throughout this review, most strategies in the past 5 years were focused on T5αH. This is because this enzyme catalyzes the most important bottleneck of the pathway. With a taxadiene conversion rate of less than 10%, and a product profile comprising several monooxygenated and few dioxygenated taxanes, it’s not surprising that strategies that aimed to increase supply of precursors, or those aiming to improve catalytic activity did not achieve much improvements since this is a major branching point of the pathway that splits flux into several off-target taxanes. The successes recorded through optimized expression (operon constructs, use of low strength promoters and low copy plasmids), a clever approach to couple expression of the protein to cell envelope stress through rSFPs, compartmentalized engineering in chloroplasts of plant cells and engineering of TXS to favor iso-taxadiene product that proved to exclusively lead to T-5α-ol are approaches that we expect to be further developed in the near future, utilizing such enabling tools as the CRISPR/Cas9 toolkit developed for *S. cerevisiae*. *Nicotiana benthamiana*, a high biomass plant with available technologies for DNA manipulation and agrobacterium-mediated transient expression systems is very promising as a chassis for heterologous expression of the Taxol pathway and is increasingly being favored for production of many other terpenoids. The Taxol pathway that comprises nine CYP450s that trigger membrane envelope stress on their expression in host cells as demonstrated with T5αH, is a very good candidate for expression in *N. benthamiana*.

Advances is high throughput sequencing technologies have enabled generation of several Taxol-related transcriptomes, and recent mining of these publicly available resources have led to isolation of two missing genes, PCL and T2′αH, though wide testing of PCL in different heterologous hosts is yet to be conclusive. Dozens of lead candidate genes for the missing steps were identified through mining transcriptomes, and with more screening platforms being established, we anticipate full elucidation of the pathway in the near future. Furthermore, we anticipate gas phase QM/MM calculations and experimental methods to reveal the mechanism of catalysis of T5αH that will guide protein engineering to overcome the bottleneck, as was done for TXS and DBAT. As sequencing technology advances in the coming years, we also expect a high-resolution genome of *Taxus* species to be assembled that will complement transcriptome data sets and accelerate gene discovery of the remaining CYP450s, PCL, and an epoxidase. Synthetic biology tools are expected to play an increasingly important role in enzyme discovery, construction and optimization of the pathway in different chassis and silencing of competing pathways.

## Author Contributions

IM and JL drafted the manuscript with support from FX. IM, JL, FX, and YW revised the manuscript. YW supervised this work. All authors contributed to the article and approved the submitted version.

## Conflict of Interest

The authors declare that the research was conducted in the absence of any commercial or financial relationships that could be construed as a potential conflict of interest.
